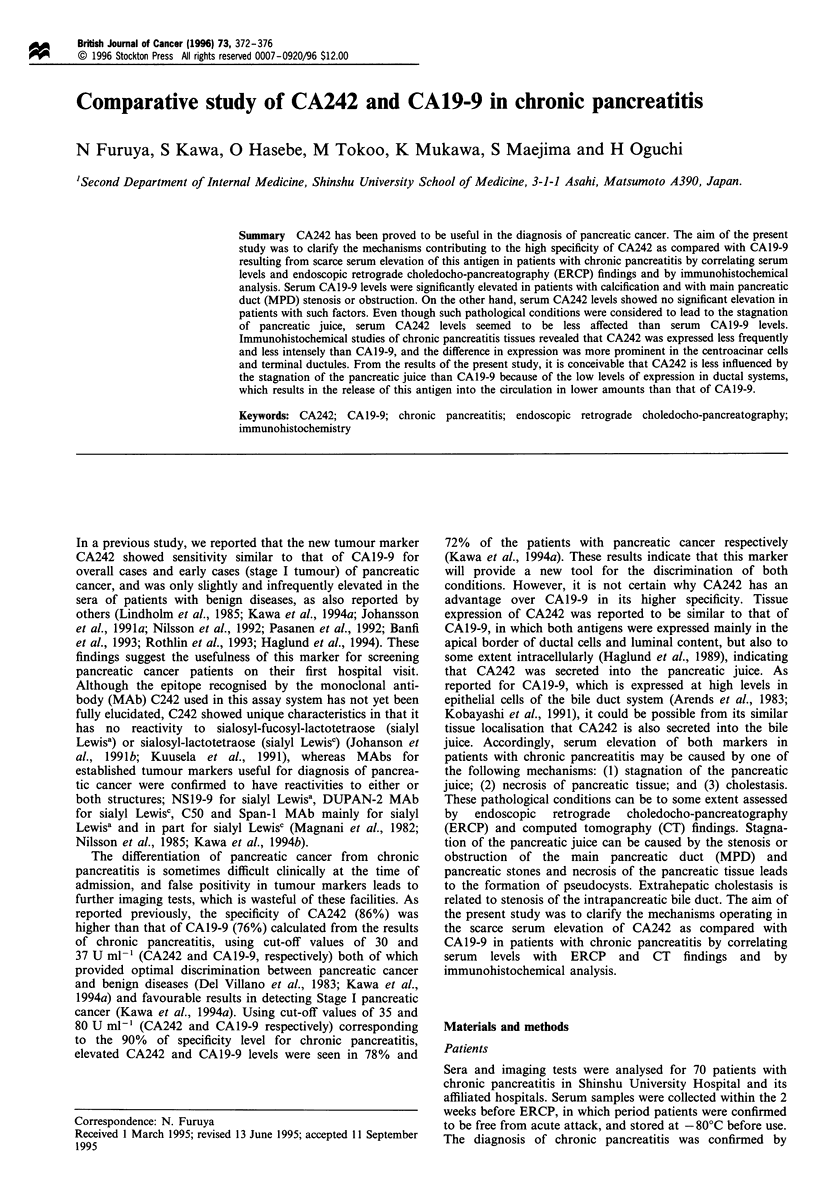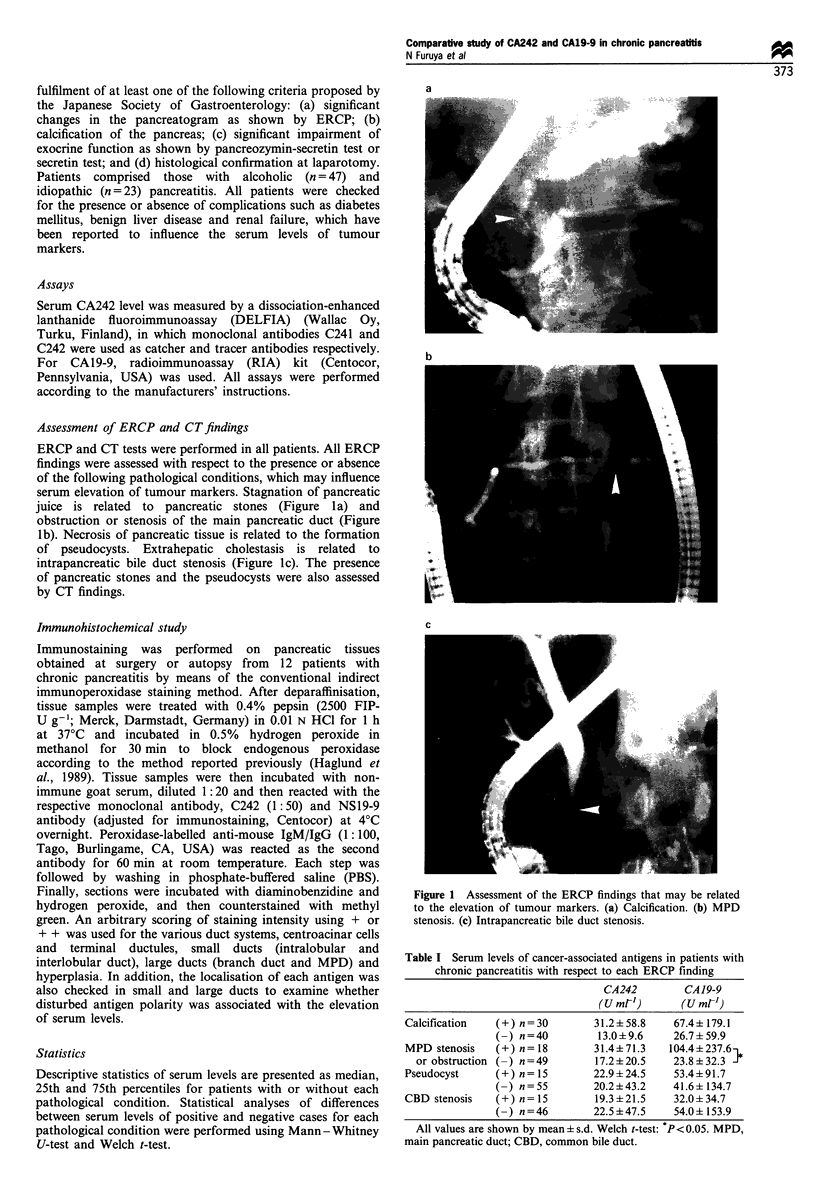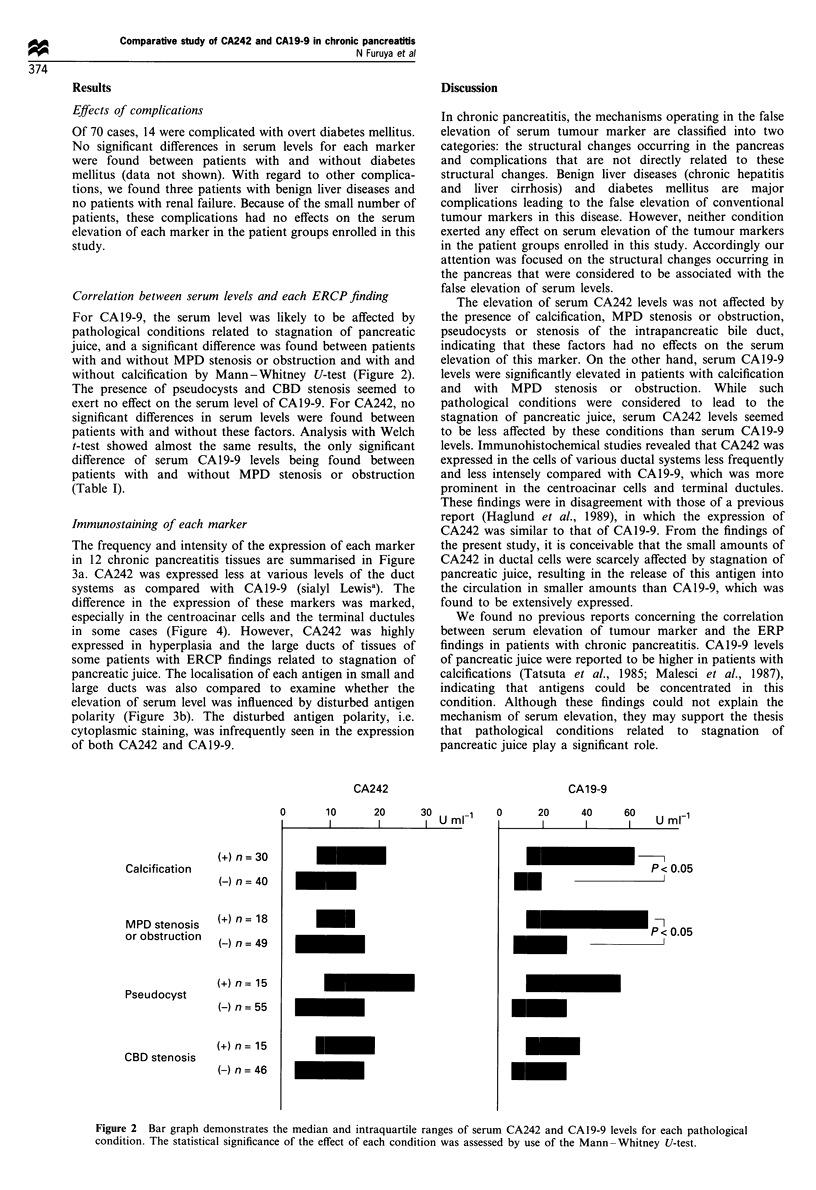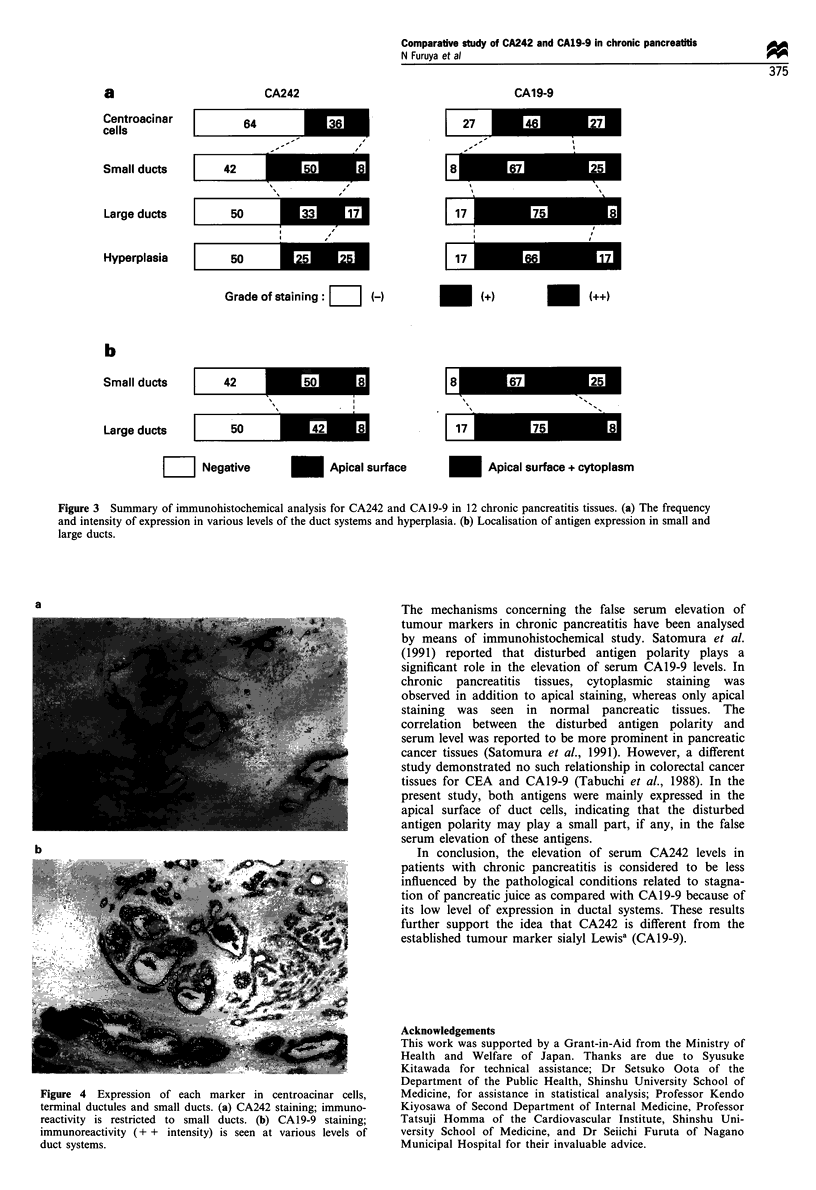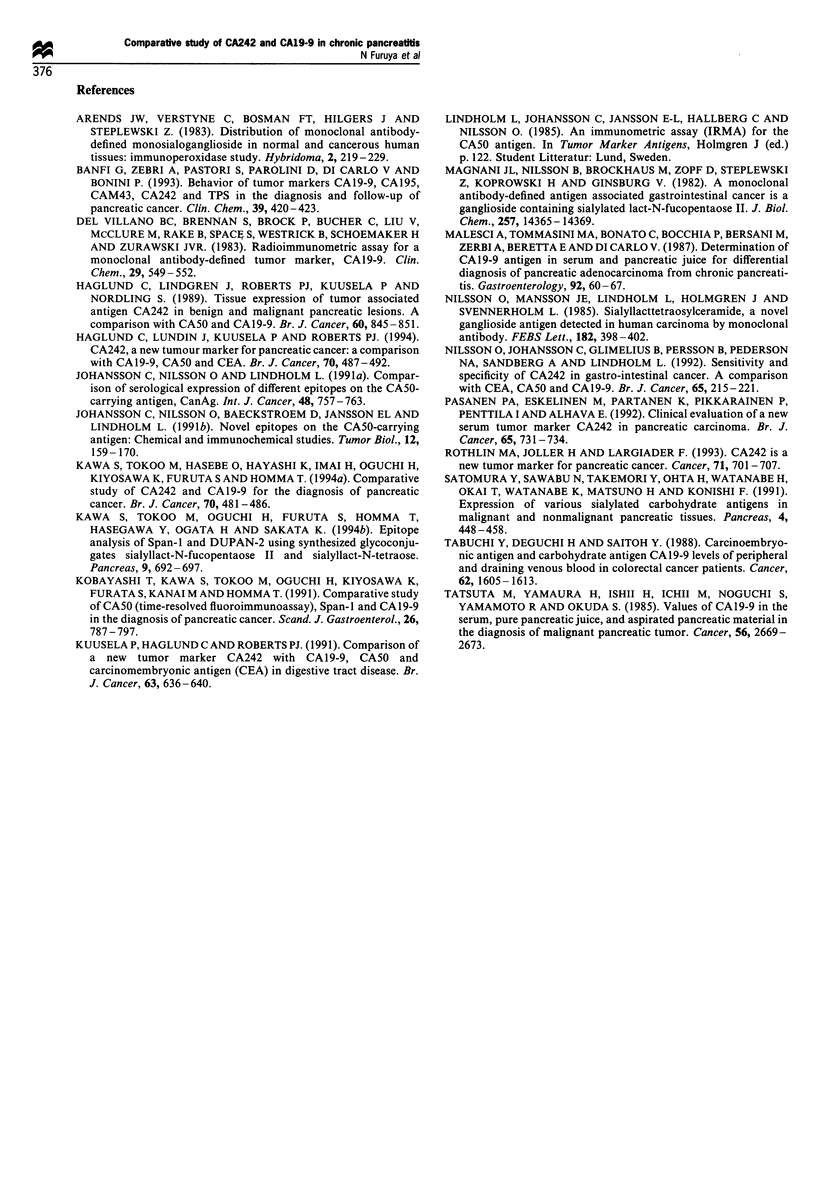# Comparative study of CA242 and CA19-9 in chronic pancreatitis.

**DOI:** 10.1038/bjc.1996.63

**Published:** 1996-02

**Authors:** N. Furuya, S. Kawa, O. Hasebe, M. Tokoo, K. Mukawa, S. Maejima, H. Oguchi

**Affiliations:** Second Department of Internal Medicine, Shinshu University School of Medicine, Matsumoto, Japan.

## Abstract

**Images:**


British Journal of Cancer (1996) 73, 372-376
© 1996 Stockton Press All rights reserved 0007-0920/96 $12.00

### Comparative study of CA242 and CA19-9 in chronic pancreatitis

N Furuya, S Kawa, O Hasebe, M Tokoo, K Mukawa, S Maejima and H Oguchi

[1]*Second Department of Internal Medicine, Shinshu University School of Medicine, 3-1-1 Asahi, Matsumoto A390, Japan.*

**Summary** CA242 has been proved to be useful in the diagnosis of pancreatic cancer. The aim of the present study was to clarify the mechanisms contributing to the high specificity of CA242 as compared with CA19-9 resulting from scarce serum elevation of this antigen in patients with chronic pancreatitis by correlating serum levels and endoscopic retrograde choledocho-pancreatography (ERCP) findings and by immunohistochemical analysis. Serum CA19-9 levels were significantly elevated in patients with calcification and with main pancreatic duct (MPD) stenosis or obstruction. On the other hand, serum CA242 levels showed no significant elevation in patients with such factors. Even though such pathological conditions were considered to lead to the stagnation of pancreatic juice, serum CA242 levels seemed to be less affected than serum CA19-9 levels. Immunohistochemical studies of chronic pancreatitis tissues revealed that CA242 was expressed less frequently and less intensely than CA19-9, and the difference in expression was more prominent in the centroacinar cells and terminal ductules. From the results of the present study, it is conceivable that CA242 is less influenced by the stagnation of the pancreatic juice than CA19-9 because of the low levels of expression in ductal systems, which results in the release of this antigen into the circulation in lower amounts than that of CA19-9.

**Keywords:** CA242; CA19-9; chronic pancreatitis; endoscopic retrograde choledocho-pancreatography; immunohistochemistry

In a previous study, we reported that the new tumour marker CA242 showed sensitivity similar to that of CA19-9 for overall cases and early cases (stage I tumour) of pancreatic cancer, and was only slightly and infrequently elevated in the sera of patients with benign diseases, as also reported by others (Lindholm *et al.*, 1985; Kawa *et al.*, 1994*a*; Johansson *et al.*, 1991*a*; Nilsson *et al.*, 1992; Pasanen *et al.*, 1992; Banfi *et al.*, 1993; Rothlin *et al.*, 1993; Haglund *et al.*, 1994). These findings suggest the usefulness of this marker for screening pancreatic cancer patients on their first hospital visit. Although the epitope recognised by the monoclonal antibody (MAb) C242 used in this assay system has not yet been fully elucidated, C242 showed unique characteristics in that it has no reactivity to sialosyl-fucosyl-lactotetraose (sialyl Lewis[a]) or sialosyl-lactotetraose (sialyl Lewis[c]) (Johanson *et al.*, 1991*b*; Kuusela *et al.*, 1991), whereas MAbs for established tumour markers useful for diagnosis of pancreatic cancer were confirmed to have reactivities to either or both structures; NS19-9 for sialyl Lewis[a], DUPAN-2 MAb for sialyl Lewis[c], C50 and Span-1 MAb mainly for sialyl Lewis[a] and in part for sialyl Lewis[c] (Magnani *et al.*, 1982; Nilsson *et al.*, 1985; Kawa *et al.*, 1994*b*).

The differentiation of pancreatic cancer from chronic pancreatitis is sometimes difficult clinically at the time of admission, and false positivity in tumour markers leads to further imaging tests, which is wasteful of these facilities. As reported previously, the specificity of CA242 (86%) was higher than that of CA19-9 (76%) calculated from the results of chronic pancreatitis, using cut-off values of 30 and 37 U $ml^{-1}$ (CA242 and CA19-9, respectively) both of which provided optimal discrimination between pancreatic cancer and benign diseases (Del Villano *et al.*, 1983; Kawa *et al.*, 1994*a*) and favourable results in detecting Stage I pancreatic cancer (Kawa *et al.*, 1994*a*). Using cut-off values of 35 and 80 U $ml^{-1}$ (CA242 and CA19-9 respectively) corresponding to the 90% of specificity level for chronic pancreatitis, elevated CA242 and CA19-9 levels were seen in 78% and 72% of the patients with pancreatic cancer respectively (Kawa *et al.*, 1994*a*). These results indicate that this marker will provide a new tool for the discrimination of both conditions. However, it is not certain why CA242 has an advantage over CA19-9 in its higher specificity. Tissue expression of CA242 was reported to be similar to that of CA19-9, in which both antigens were expressed mainly in the apical border of ductal cells and luminal content, but also to some extent intracellularly (Haglund *et al.*, 1989), indicating that CA242 was secreted into the pancreatic juice. As reported for CA19-9, which is expressed at high levels in epithelial cells of the bile duct system (Arends *et al.*, 1983; Kobayashi *et al.*, 1991), it could be possible from its similar tissue localisation that CA242 is also secreted into the bile juice. Accordingly, serum elevation of both markers in patients with chronic pancreatitis may be caused by one of the following mechanisms: (1) stagnation of the pancreatic juice; (2) necrosis of pancreatic tissue; and (3) cholestasis. These pathological conditions can be to some extent assessed by endoscopic retrograde choledocho-pancreatography (ERCP) and computed tomography (CT) findings. Stagnation of the pancreatic juice can be caused by the stenosis or obstruction of the main pancreatic duct (MPD) and pancreatic stones and necrosis of the pancreatic tissue leads to the formation of pseudocysts. Extrahepatic cholestasis is related to stenosis of the intrapancreatic bile duct. The aim of the present study was to clarify the mechanisms operating in the scarce serum elevation of CA242 as compared with CA19-9 in patients with chronic pancreatitis by correlating serum levels with ERCP and CT findings and by immunohistochemical analysis.

### Materials and methods

### *Patients*

Sera and imaging tests were analysed for 70 patients with chronic pancreatitis in Shinshu University Hospital and its affiliated hospitals. Serum samples were collected within the 2 weeks before ERCP, in which period patients were confirmed to be free from acute attack, and stored at −80°C before use. The diagnosis of chronic pancreatitis was confirmed by

Correspondence: N. Furuya
Received 1 March 1995; revised 13 June 1995; accepted 11 September 1995

fulfilment of at least one of the following criteria proposed by the Japanese Society of Gastroenterology: (a) significant changes in the pancreatogram as shown by ERCP; (b) calcification of the pancreas; (c) significant impairment of exocrine function as shown by pancreozymin-secretin test or secretin test; and (d) histological confirmation at laparotomy. Patients comprised those with alcoholic ($n=47$) and idiopathic ($n=23$) pancreatitis. All patients were checked for the presence or absence of complications such as diabetes mellitus, benign liver disease and renal failure, which have been reported to influence the serum levels of tumour markers.

*Assays*

Serum CA242 level was measured by a dissociation-enhanced lanthanide fluoroimmunoassay (DELFIA) (Wallac Oy, Turku, Finland), in which monoclonal antibodies C241 and C242 were used as catcher and tracer antibodies respectively. For CA19-9, radioimmunoassay (RIA) kit (Centocor, Pennsylvania, USA) was used. All assays were performed according to the manufacturers' instructions.

*Assessment of ERCP and CT findings*

ERCP and CT tests were performed in all patients. All ERCP findings were assessed with respect to the presence or absence of the following pathological conditions, which may influence serum elevation of tumour markers. Stagnation of pancreatic juice is related to pancreatic stones (Figure 1a) and obstruction or stenosis of the main pancreatic duct (Figure 1b). Necrosis of pancreatic tissue is related to the formation of pseudocysts. Extrahepatic cholestasis is related to intrapancreatic bile duct stenosis (Figure 1c). The presence of pancreatic stones and the pseudocysts were also assessed by CT findings.

*Immunohistochemical study*

Immunostaining was performed on pancreatic tissues obtained at surgery or autopsy from 12 patients with chronic pancreatitis by means of the conventional indirect immunoperoxidase staining method. After deparaffinisation, tissue samples were treated with 0.4% pepsin (2500 FIP-U $g^{-1}$; Merck, Darmstadt, Germany) in 0.01 N HCl for 1 h at 37°C and incubated in 0.5% hydrogen peroxide in methanol for 30 min to block endogenous peroxidase according to the method reported previously (Haglund *et al.*, 1989). Tissue samples were then incubated with non-immune goat serum, diluted 1:20 and then reacted with the respective monoclonal antibody, C242 (1:50) and NS19-9 antibody (adjusted for immunostaining, Centocor) at 4°C overnight. Peroxidase-labelled anti-mouse IgM/IgG (1:100, Tago, Burlingame, CA, USA) was reacted as the second antibody for 60 min at room temperature. Each step was followed by washing in phosphate-buffered saline (PBS). Finally, sections were incubated with diaminobenzidine and hydrogen peroxide, and then counterstained with methyl green. An arbitrary scoring of staining intensity using + or + + was used for the various duct systems, centroacinar cells and terminal ductules, small ducts (intralobular and interlobular duct), large ducts (branch duct and MPD) and hyperplasia. In addition, the localisation of each antigen was also checked in small and large ducts to examine whether disturbed antigen polarity was associated with the elevation of serum levels.

*Statistics*

Descriptive statistics of serum levels are presented as median, 25th and 75th percentiles for patients with or without each pathological condition. Statistical analyses of differences between serum levels of positive and negative cases for each pathological condition were performed using Mann–Whitney *U*-test and Welch *t*-test.

**Figure 1** Assessment of the ERCP findings that may be related to the elevation of tumour markers. (**a**) Calcification. (**b**) MPD stenosis. (**c**) Intrapancreatic bile duct stenosis.

**Table I** Serum levels of cancer-associated antigens in patients with chronic pancreatitis with respect to each ERCP finding

| | | *CA242* ($U\ ml^{-1}$) | *CA19-9* ($U\ ml^{-1}$) |
|---|---|---|---|
| Calcification | (+) $n=30$ | 31.2 ± 58.8 | 67.4 ± 179.1 |
| | (−) $n=40$ | 13.0 ± 9.6 | 26.7 ± 59.9 |
| MPD stenosis | (+) $n=18$ | 31.4 ± 71.3 | 104.4 ± 237.6 ]* |
| or obstruction | (−) $n=49$ | 17.2 ± 20.5 | 23.8 ± 32.3 |
| Pseudocyst | (+) $n=15$ | 22.9 ± 24.5 | 53.4 ± 91.7 |
| | (−) $n=55$ | 20.2 ± 43.2 | 41.6 ± 134.7 |
| CBD stenosis | (+) $n=15$ | 19.3 ± 21.5 | 32.0 ± 34.7 |
| | (−) $n=46$ | 22.5 ± 47.5 | 54.0 ± 153.9 |

All values are shown by mean ± s.d. Welch *t*-test: $^{*}P<0.05$. MPD, main pancreatic duct; CBD, common bile duct.

### Results

### *Effects of complications*

Of 70 cases, 14 were complicated with overt diabetes mellitus. No significant differences in serum levels for each marker were found between patients with and without diabetes mellitus (data not shown). With regard to other complications, we found three patients with benign liver diseases and no patients with renal failure. Because of the small number of patients, these complications had no effects on the serum elevation of each marker in the patient groups enrolled in this study.

### *Correlation between serum levels and each ERCP finding*

For CA19-9, the serum level was likely to be affected by pathological conditions related to stagnation of pancreatic juice, and a significant difference was found between patients with and without MPD stenosis or obstruction and with and without calcification by Mann–Whitney *U*-test (Figure 2). The presence of pseudocysts and CBD stenosis seemed to exert no effect on the serum level of CA19-9. For CA242, no significant differences in serum levels were found between patients with and without these factors. Analysis with Welch *t*-test showed almost the same results, the only significant difference of serum CA19-9 levels being found between patients with and without MPD stenosis or obstruction (Table I).

### *Immunostaining of each marker*

The frequency and intensity of the expression of each marker in 12 chronic pancreatitis tissues are summarised in Figure 3a. CA242 was expressed less at various levels of the duct systems as compared with CA19-9 (sialyl Lewis[a]). The difference in the expression of these markers was marked, especially in the centroacinar cells and the terminal ductules in some cases (Figure 4). However, CA242 was highly expressed in hyperplasia and the large ducts of tissues of some patients with ERCP findings related to stagnation of pancreatic juice. The localisation of each antigen in small and large ducts was also compared to examine whether the elevation of serum level was influenced by disturbed antigen polarity (Figure 3b). The disturbed antigen polarity, i.e. cytoplasmic staining, was infrequently seen in the expression of both CA242 and CA19-9.

### Discussion

In chronic pancreatitis, the mechanisms operating in the false elevation of serum tumour marker are classified into two categories: the structural changes occurring in the pancreas and complications that are not directly related to these structural changes. Benign liver diseases (chronic hepatitis and liver cirrhosis) and diabetes mellitus are major complications leading to the false elevation of conventional tumour markers in this disease. However, neither condition exerted any effect on serum elevation of the tumour markers in the patient groups enrolled in this study. Accordingly our attention was focused on the structural changes occurring in the pancreas that were considered to be associated with the false elevation of serum levels.

The elevation of serum CA242 levels was not affected by the presence of calcification, MPD stenosis or obstruction, pseudocysts or stenosis of the intrapancreatic bile duct, indicating that these factors had no effects on the serum elevation of this marker. On the other hand, serum CA19-9 levels were significantly elevated in patients with calcification and with MPD stenosis or obstruction. While such pathological conditions were considered to lead to the stagnation of pancreatic juice, serum CA242 levels seemed to be less affected by these conditions than serum CA19-9 levels. Immunohistochemical studies revealed that CA242 was expressed in the cells of various ductal systems less frequently and less intensely compared with CA19-9, which was more prominent in the centroacinar cells and terminal ductules. These findings were in disagreement with those of a previous report (Haglund *et al.*, 1989), in which the expression of CA242 was similar to that of CA19-9. From the findings of the present study, it is conceivable that the small amounts of CA242 in ductal cells were scarcely affected by stagnation of pancreatic juice, resulting in the release of this antigen into the circulation in smaller amounts than CA19-9, which was found to be extensively expressed.

We found no previous reports concerning the correlation between serum elevation of tumour marker and the ERP findings in patients with chronic pancreatitis. CA19-9 levels of pancreatic juice were reported to be higher in patients with calcifications (Tatsuta *et al.*, 1985; Malesci *et al.*, 1987), indicating that antigens could be concentrated in this condition. Although these findings could not explain the mechanism of serum elevation, they may support the thesis that pathological conditions related to stagnation of pancreatic juice play a significant role.

**Figure 2** Bar graph demonstrates the median and intraquartile ranges of serum CA242 and CA19-9 levels for each pathological condition. The statistical significance of the effect of each condition was assessed by use of the Mann–Whitney *U*-test.

**Figure 3** Summary of immunohistochemical analysis for CA242 and CA19-9 in 12 chronic pancreatitis tissues. (**a**) The frequency and intensity of expression in various levels of the duct systems and hyperplasia. (**b**) Localisation of antigen expression in small and large ducts.

**Figure 4** Expression of each marker in centroacinar cells, terminal ductules and small ducts. (**a**) CA242 staining; immunoreactivity is restricted to small ducts. (**b**) CA19-9 staining; immunoreactivity (+ + intensity) is seen at various levels of duct systems.

The mechanisms concerning the false serum elevation of tumour markers in chronic pancreatitis have been analysed by means of immunohistochemical study. Satomura *et al.* (1991) reported that disturbed antigen polarity plays a significant role in the elevation of serum CA19-9 levels. In chronic pancreatitis tissues, cytoplasmic staining was observed in addition to apical staining, whereas only apical staining was seen in normal pancreatic tissues. The correlation between the disturbed antigen polarity and serum level was reported to be more prominent in pancreatic cancer tissues (Satomura *et al.*, 1991). However, a different study demonstrated no such relationship in colorectal cancer tissues for CEA and CA19-9 (Tabuchi *et al.*, 1988). In the present study, both antigens were mainly expressed in the apical surface of duct cells, indicating that the disturbed antigen polarity may play a small part, if any, in the false serum elevation of these antigens.

In conclusion, the elevation of serum CA242 levels in patients with chronic pancreatitis is considered to be less influenced by the pathological conditions related to stagnation of pancreatic juice as compared with CA19-9 because of its low level of expression in ductal systems. These results further support the idea that CA242 is different from the established tumour marker sialyl Lewis[a] (CA19-9).

**Acknowledgements**
This work was supported by a Grant-in-Aid from the Ministry of Health and Welfare of Japan. Thanks are due to Syusuke Kitawada for technical assistance; Dr Setsuko Oota of the Department of the Public Health, Shinshu University School of Medicine, for assistance in statistical analysis; Professor Kendo Kiyosawa of Second Department of Internal Medicine, Professor Tatsuji Homma of the Cardiovascular Institute, Shinshu University School of Medicine, and Dr Seiichi Furuta of Nagano Municipal Hospital for their invaluable advice.

### References

ARENDS JW, VERSTYNE C, BOSMAN FT, HILGERS J AND STEPLEWSKI Z. (1983). Distribution of monoclonal antibody-defined monosialoganglioside in normal and cancerous human tissues: immunoperoxidase study. *Hybridoma*, **2**, 219–229.

BANFI G, ZEBRI A, PASTORI S, PAROLINI D, DI CARLO V AND BONINI P. (1993). Behavior of tumor markers CA19-9, CA195, CAM43, CA242 and TPS in the diagnosis and follow-up of pancreatic cancer. *Clin. Chem.*, **39**, 420–423.

DEL VILLANO BC, BRENNAN S, BROCK P, BUCHER C, LIU V, McCLURE M, RAKE B, SPACE S, WESTRICK B, SCHOEMAKER H AND ZURAWSKI JVR. (1983). Radioimmunometric assay for a monoclonal antibody-defined tumor marker, CA19-9. *Clin. Chem.*, **29**, 549–552.

HAGLUND C, LINDGREN J, ROBERTS PJ, KUUSELA P AND NORDLING S. (1989). Tissue expression of tumor associated antigen CA242 in benign and malignant pancreatic lesions. A comparison with CA50 and CA19-9. *Br. J. Cancer*, **60**, 845–851.

HAGLUND C, LUNDIN J, KUUSELA P AND ROBERTS PJ. (1994). CA242, a new tumour marker for pancreatic cancer: a comparison with CA19-9, CA50 and CEA. *Br. J. Cancer*, **70**, 487–492.

JOHANSSON C, NILSSON O AND LINDHOLM L. (1991*a*). Comparison of serological expression of different epitopes on the CA50-carrying antigen, CanAg. *Int. J. Cancer*, **48**, 757–763.

JOHANSSON C, NILSSON O, BAECKSTROEM D, JANSSON EL AND LINDHOLM L. (1991*b*). Novel epitopes on the CA50-carrying antigen: Chemical and immunochemical studies. *Tumor Biol.*, **12**, 159–170.

KAWA S, TOKOO M, HASEBE O, HAYASHI K, IMAI H, OGUCHI H, KIYOSAWA K, FURUTA S AND HOMMA T. (1994*a*). Comparative study of CA242 and CA19-9 for the diagnosis of pancreatic cancer. *Br. J. Cancer*, **70**, 481–486.

KAWA S, TOKOO M, OGUCHI H, FURUTA S, HOMMA T, HASEGAWA Y, OGATA H AND SAKATA K. (1994*b*). Epitope analysis of Span-1 and DUPAN-2 using synthesized glycoconjugates sialyllact-N-fucopentaose II and sialyllact-N-tetraose. *Pancreas*, **9**, 692–697.

KOBAYASHI T, KAWA S, TOKOO M, OGUCHI H, KIYOSAWA K, FURATA S, KANAI M AND HOMMA T. (1991). Comparative study of CA50 (time-resolved fluoroimmunoassay), Span-1 and CA19-9 in the diagnosis of pancreatic cancer. *Scand. J. Gastroenterol.*, **26**, 787–797.

KUUSELA P, HAGLUND C AND ROBERTS PJ. (1991). Comparison of a new tumor marker CA242 with CA19-9, CA50 and carcinomembryonic antigen (CEA) in digestive tract disease. *Br. J. Cancer*, **63**, 636–640.

LINDHOLM L, JOHANSSON C, JANSSON E-L, HALLBERG C AND NILSSON O. (1985). An immunometric assay (IRMA) for the CA50 antigen. In *Tumor Marker Antigens*, Holmgren J (ed.) p. 122. Student Litteratur: Lund, Sweden.

MAGNANI JL, NILSSON B, BROCKHAUS M, ZOPF D, STEPLEWSKI Z, KOPROWSKI H AND GINSBURG V. (1982). A monoclonal antibody-defined antigen associated gastrointestinal cancer is a ganglioside containing sialylated lact-N-fucopentaose II. *J. Biol. Chem.*, **257**, 14365–14369.

MALESCI A, TOMMASINI MA, BONATO C, BOCCHIA P, BERSANI M, ZERBI A, BERETTA E AND DI CARLO V. (1987). Determination of CA19-9 antigen in serum and pancreatic juice for differential diagnosis of pancreatic adenocarcinoma from chronic pancreatitis. *Gastroenterology*, **92**, 60–67.

NILSSON O, MANSSON JE, LINDHOLM L, HOLMGREN J AND SVENNERHOLM L. (1985). Sialyllacttetraosylceramide, a novel ganglioside antigen detected in human carcinoma by monoclonal antibody. *FEBS Lett.*, **182**, 398–402.

NILSSON O, JOHANSSON C, GLIMELIUS B, PERSSON B, PEDERSON NA, SANDBERG A AND LINDHOLM L. (1992). Sensitivity and specificity of CA242 in gastro-intestinal cancer. A comparison with CEA, CA50 and CA19-9. *Br. J. Cancer*, **65**, 215–221.

PASANEN PA, ESKELINEN M, PARTANEN K, PIKKARAINEN P, PENTTILA I AND ALHAVA E. (1992). Clinical evaluation of a new serum tumor marker CA242 in pancreatic carcinoma. *Br. J. Cancer*, **65**, 731–734.

ROTHLIN MA, JOLLER H AND LARGIADER F. (1993). CA242 is a new tumor marker for pancreatic cancer. *Cancer*, **71**, 701–707.

SATOMURA Y, SAWABU N, TAKEMORI Y, OHTA H, WATANABE H, OKAI T, WATANABE K, MATSUNO H AND KONISHI F. (1991). Expression of various sialylated carbohydrate antigens in malignant and nonmalignant pancreatic tissues. *Pancreas*, **4**, 448–458.

TABUCHI Y, DEGUCHI H AND SAITOH Y. (1988). Carcinoembryonic antigen and carbohydrate antigen CA19-9 levels of peripheral and draining venous blood in colorectal cancer patients. *Cancer*, **62**, 1605–1613.

TATSUTA M, YAMAURA H, ISHII H, ICHII M, NOGUCHI S, YAMAMOTO R AND OKUDA S. (1985). Values of CA19-9 in the serum, pure pancreatic juice, and aspirated pancreatic material in the diagnosis of malignant pancreatic tumor. *Cancer*, **56**, 2669–2673.